# 

**Published:** 1883-07

**Authors:** 


### CONTENTS:

| | PAGE. |
|---|---|
| **Original Communications:** | |
| Aphasia. By H. R. Hopkins, M. D., | 529 |
| Pares s, or General Paralysis of the Insane. By Wm. D. Granger, M.D., | 537 |
| **Selections:** | |
| Convulsions in Children, | 546 |
| Infantile Leucorrhœa, | 550 |
| Rule for Examination of Urine, | 553 |
| Adulteration of Brandy, | 554 |
| **Society Reports:** | |
| Buffalo Medical and Surgical Association, | 555 |
| Erie County Medical Society, | 557 |
| **Editorial:** | |
| Something Worth Fighting for, | 563 |
| The American Medical Association, | 565 |
| The Committ:e on Legislation, | 566 |
| **Reviews:** | |
| A Manual of Histology, | 567 |
| A Practical Treatise on Impotence, Sterility and Allied Disorders of the Male Sexual Organs, | 568 |
| A Treatise on Therapeutics, Comprising Materia Medica and Toxicology, with Especial Reference to the Application of the Physiological Action of Drugs to Clinical Medicine, | 568 |

